# Automated Field-of-View, Illumination, and Recognition Algorithm Design of a Vision System for Pick-and-Place Considering Colour Information in Illumination and Images

**DOI:** 10.3390/s18051656

**Published:** 2018-05-22

**Authors:** Yibing Chen, Taiki Ogata, Tsuyoshi Ueyama, Toshiyuki Takada, Jun Ota

**Affiliations:** 1 Department of Precision Engineering, the University of Tokyo, Tokyo 113-8656, Japan; y.chen@race.u-tokyo.ac.jp; 2 Research into Artifacts, Center for Engineering (RACE), The University of Tokyo, Chiba 277-8568, Japan; ota@race.u-tokyo.ac.jp; 3 Denso Wave Incorporated, Aichi 470-2298, Japan; tsuyoshi.ueyama@denso-wave.co.jp (T.U.); toshiyuki.takada@denso-wave.co.jp (T.T.)

**Keywords:** automated design, vision system, FOV, illumination, recognition algorithm

## Abstract

Machine vision is playing an increasingly important role in industrial applications, and the automated design of image recognition systems has been a subject of intense research. This study has proposed a system for automatically designing the field-of-view (FOV) of a camera, the illumination strength and the parameters in a recognition algorithm. We formulated the design problem as an optimisation problem and used an experiment based on a hierarchical algorithm to solve it. The evaluation experiments using translucent plastics objects showed that the use of the proposed system resulted in an effective solution with a wide FOV, recognition of all objects and 0.32 mm and 0.4° maximal positional and angular errors when all the RGB (red, green and blue) for illumination and R channel image for recognition were used. Though all the RGB illumination and grey scale images also provided recognition of all the objects, only a narrow FOV was selected. Moreover, full recognition was not achieved by using only G illumination and a grey-scale image. The results showed that the proposed method can automatically design the FOV, illumination and parameters in the recognition algorithm and that tuning all the RGB illumination is desirable even when single-channel or grey-scale images are used for recognition.

## 1. Introduction

Machine vision technologies have been widely applied in the industrial field for automated visual inspection, process control, parts identification, and robotic guidance [1,2]. Designers have been attempting to tune the parameters for a variety of vision systems. A vision system is usually composed of a camera and an illumination and recognition algorithm [3], which are also known as the main design factors of a vision system. In the object recognition system of a pick-and-place robot, for example, the camera position needs to be set to obtain a suitable Field-of-View (hereinafter referred to as FOV), the illumination requires to be changed to strengthen features in targets, and the image recognition process needs to be optimised through parameter tuning. As this creates a number of conflicting variables, the design process must be reiterated until acceptable results are obtained. This is a time-consuming task even when carried out by experts, and even a simple pick-and-place vision system usually takes several days to design.

Previous studies have addressed the automated design of sensor locations [4,5,6,7,8,9,10,11,12], illumination levels [13,14,15,16,17,18], and recognition algorithms [19,20,21,22,23,24,25]. Some studies proposed a method to automatically determine the place to set a vision sensor for specific features of recognition targets to satisfy the specific constraints of recognition requirements [4,5,6]. Some other studies focused on the sensing strategies for recognition and localisation of targets with the help of 3D models [7,8,9]. Moreover, several sensor planning methods were designed respectively based on the vision tasks in [10,11,12].

Researches on automated planning of illumination parameters have also been carried out. Experiment-based approaches have been proposed to optimise illumination with a set of images of an object captured under different illumination conditions [13,14]. Besides, illumination planning methods based on mathematical models of illumination were proposed [15,16]. More recently, with the help of rendering techniques, illumination planning approaches based on computer simulation were reported [17,18].

Some studies have attempted to automate the image processing procedures. Automated image pre-processing techniques were proposed in [19,20,21]. Some other studies investigated the automated design for feature extraction [22,23]. Automated generation of discriminators were discussed in [24,25]. Especially, an approach was proposed for automatically designing an image recognition procedure from the aspect of pre-processing, feature extraction, and discriminator [25].

It is clear from the former studies that an overall design approach to vision systems could hardly be found. One reason is probably the interactions among the different design factors. Therefore, in the case of an overall design, the situation becomes more and more complex because design factors influence each other in unpredictable ways. To the best of our knowledge, a design approach to deal with various design factors has been presented only in [26,27]. Experiment-based methods were applied to achieve an automated design of a vision system on the basis of illumination and a recognition algorithm in [26]. By adding FOV, Chen Y., et al. [27] provided a more comprehensive vision system design method. The problem is that in both the studies, the recognition tasks were far from a being practical task because only one or two objects were considered.

Another obstacle in taking out an overall design approach consists of the uncertainties of the real world. Colour is known as one of the uncertainties in image recognition. Because objects’ colours change with illumination, colour- and illumination-invariant recognition methods have been postulated [28,29,30,31]. The greyscale process transforms colourful multi-channel images into grey and single-channel ones, which could be more easily understood by vision systems. Such multi-channel image encoding approaches were presented in [32,33,34], while it was also pointed out that greyscale approaches could influence the recognition performance to a great extent [35]. In this study, we have mainly focused on the uncertainties caused by colour information contained in both illumination and grabbed images.

This study transferred a vision system design problem into an optimisation problem and proposed an experiment-based approach to realise an automated vision system design. It was proved in the study that the proposed design could provide vision systems that were effective in pick-and-place tasks with suitable parameters of the FOV, illumination and recognition algorithm. Moreover, we studied one kind of uncertainties from the real world, that is, colour information illuminate from the light source that is absorbed by the camera sensor. Thus, we conducted an experiment of automated designs using our proposed method by changing the colour channels that were utilised for both illumination and recognition. By this experiment, we investigated: (1) whether or not providing colourful illumination improves recognition accuracy when even the vision system reads only the greyscale images and (2) whether or not single-channel images like R-channel images provide better performance in recognition than the greyscale ones.

## 2. Problem Formulation

### 2.1. Preconditions

The vision systems applied to a pick-and-place robot are set to the design target in this study. In order to pick objects and place them in the right positions, the vision systems are required to provide the following information:

● *Types*

Before picking up an object, the system must know which kind of object to select. For instance, in some sorting tasks, type refers to information that describes targets’ appearance, such as the shape, colour, and which side is facing upwards. By using the type information, the robot is able to distinguish the target objects into several categories. Therefore, a dictionary which contains type information must be available to vision systems for pick-and-place.

● *Position*

For a pick-and-place robot, definitely ‘pick’ is one of the most important quests. To pick objects up, position information, in other words, the centre of gravity of each target object should be identified. A vision system captures positional information in pixels. In this study, the coordinate origin is set to the left-top of the image, the x-axis forward direction to the right, and the y-axial forward direction is downward.

● *Orientations*

For both ‘pick’ and ‘place’ quests, the orientation information is important. That is to say, the vision system must also provide angular information about each object. In this study, we assumed the measurement range to be [0, 360).

To clarify the problem, the working environment for the proposed system is set up based on the following three requirements: choice of camera, assumed scenes of recognition, and image processing software. The details for each of these requirements are given as follows:

● *Camera*

Compared to binocular cameras, monocular cameras are more widely used in pick-and-place tasks. As a result, we used a monocular CMOS (Complementary Metal Oxide Semiconductor) camera in our system. Since the camera is mounted on the end effector of an industrial manipulator, its viewpoint is held perpendicular to the workspace on which the recognition targets are arranged; the FOV is therefore of a 2D type.

● *Scenes*

Based on where to pick objects, pick-and-place tasks could be categorised into two types: tasks in dynamic systems, for example, a moving conveyor and tasks in static systems such as a tray. In this study, we chose the latter one as the option for the proposed system. In this case, all the recognition targets are placed in a limited space. This means that the camera distance at which all objects can be captured in a single image can be specified in advance. Moreover, just like most situations in industrial applications, the recognition targets are placed on the same plane surface, without overlaps. This is also true for industrial applications such as picking objects from a conveyor.

● *Software*

In this study, the proposed system was tested with a commercially available image processing library: MVTec HALCON (MVTec, Seeshaupt, Germany).

### 2.2. Design Variables

By describing the settings and the working environment, the basic information on the target vision system to be designed was provided. In order to arrive at a proper design of the described vision system, several parameters, or we can call them design variables, are required to be optimised. To further clarify the problem, such design variables are determined in this section.

In general, a vision system could be established by considering three design factors, which are: illumination condition; camera FOV; and the recognition algorithm. The illumination is usually designed for its strength and colour, illuminating the workpieces and repressing the reflections at the same time. Camera FOV determines the resolution with which the targets are recognised and the size of the recognition area. By tuning FOV, accuracy and efficiency of the vision system could be balanced. Besides, in order to maximise the performance of the chosen recognition algorithm, some parameters inside the algorithm also require optimisation.

The design variables of the vision system could also be taken as parameters of the optimisation problem that were defined in the previous section. Categorised by the three design factors, the design variables of this study are addressed as follows (details are given in Table 1):

● *FOV*

FOV is the extent of the observable world that is seen at any given moment. In the case of a camera set up for pick-and-place tasks, the FOV directly determines the number of objects that can be captured in a single image. To maximise the efficiency of recognition, the FOV must be maximised while meeting the required accuracy tolerances. In this study, FOV was balanced from the viewpoint of shoot time and camera distance.

Shoot time means the time taken by the camera to capture figures within the current camera distance. Obviously, with a limited FOV size, the vision system could not comprehend the intricate details of the workspace. Thus, it requires a system based on a moving camera which captures images several times.

On the other hand, camera distance refers to the distance from the camera lens to the plane on which the recognition targets are placed. As mentioned in the preconditions, the camera’s viewpoint is held perpendicular to the workspace; the distance therefore reflects the actual FOV size.

● *Illumination*

The illumination variables include the strength of the red, green, and blue components. Increasing the strength may produce reflections, whereas at low strength, some details of the target objects may not be captured. Both will reduce the recognition accuracy. Additionally, some details may be enhanced by selecting the specific colour of illumination. We therefore allowed the strength of each RGB component to be controlled individually. The illumination strength ranges from 0 to 255, and is searched by an increment.

● *Recognition Algorithm*

Not only the recognition algorithm but also the parameters inside the chosen algorithm influence the performance of a vision system. We just focus on the latter to optimise the inner parameters of a given recognition algorithm.

The inner parameters, for example, image pre-processing parameters or parameters for making proper templates, could more or less influence the performance of a recognition algorithm. Since different parameters may have their own properties, the optimisation method should be designed individually.

Moreover, no matter what recognition algorithm is used, a discriminator to classify correct and incorrect detections by the recognition process is required. The discriminators should also be considered as one of the design variables.

### 2.3. Inputs and Outputs

#### 2.3.1. Inputs

Aiming to turn the vision system design process into a fully-automated one, manual operations during the process of design must be minimised. Hence, the inputs to the automated design system should be considered from many aspects which are preparation data for both scenes and templates, ground truth data, and camera calibration data.

- *Preparation Data for Scenes:*
  $$S=\left(S_{1},\ldots ,\;S_{i},\ldots ,\;S_{n}\right),$$
  here, *S_i_* denotes the *i*-th coordinate on the work plane of the position where the corresponding scene was set, and *n* the total number of scenes. The number of images required to capture one scene depends on the camera distance.
  $$S_{i}=\left(x_{i},\ldots ,\;y_{i},\ldots ,\;z_{i}\right),$$
  Each *S_i_* contains the locations of *x*, *y,* and *z* directions such that the manipulator can hold the camera and capture images of the existing scene. *z_i_* describes the distance from the camera to the plane where the recognition targets are arranged.
- *Preparation Data for Templates:*
  $$T=\left(T_{1},\ldots ,\;T_{l},\ldots ,\;T_{nT}\right),$$
  where *nT* represents the total number of recognition target kinds.
  $$T_{l}=\left(x,\;y,\;z,\;x_{l},\;y_{l},\;w_{l},\;h_{l}\right),$$
  the *l*-th template is prepared by automatically cutting the object image from the original image which was obtained by holding the camera at the position (*x*, *y*, *z*). By using the position of the objects in the acquired image, namely *x_l_* and *y_l_*, as well as the predetermined width and height, *w_l_* and *h_l_*, the template could be obtained.
- *Ground Truth Data:*
  $$G=\left(G_{S_{1}},\ldots ,\;G_{S_{i}},\ldots ,\;G_{S_{n}}\right),$$
  where $G_{S_{i}}$ denotes the ground truth data for the *i*-th scene.
  $$G_{S_{i}}=\left(G_{S_{i},\;1},\ldots ,\;G_{S_{i},\;k},\ldots ,\;G_{S_{i},\;m_{i}}\right);$$
  however, the scene contains many recognition targets; the ground truth data always include information on each recognition target, from the 1st to the *m_i_*-th.
  $$G_{S_{i},\;k}=\left(Type_{i,k},\;x_{i,k},\;y_{i,k},\;\theta_{i,k}\right),$$
  the ground truth data for each object includes the object type, the *x* and *y* position in captured images and the orientation angle.
- *Camera Calibration Data:*
  $$C=\left(C_{1},\ldots ,\;C_{i},\ldots ,\;C_{n}\right),$$
  where *C_i_* denotes the *i*-th image for calibration and n the total number of images required for a calibration.

#### 2.3.2. Outputs

The system output is the optimal solution to the set of design variables.

- *Optimal solution:*
  $$S_{olution}=\left(R,\;G,\;B,P_{recognition}\right),$$
  where *R*, *G,* and *B* denote the light strength of red, green and blue, and ***P****_recognition_* the set of parameters related to the chosen recognition algorithm. Especially, ***P****_recognition_* consists of:
  $$P_{recognition}=\left(P_{1},P_{2},\;\ldots ,P_{n}\right)$$
  the entire number of parameters *n* is determined by the chosen recognition algorithm.

### 2.4. Evaluation Function and Constraints

#### 2.4.1. Evaluation Function

The evaluation uses four values which are the FOV size, *F_measure_*, positional error, and angular error.

The shoot time describes the FOV and largely determines the computing speed, as the time cost increases in line with the number of images and camera movements.

The *F_measure_* is used to describe the accuracy of recognition. It considers both the *P_recision_* and *R_ecall_*, and the definition is given by Equation (1):

(1) $$F_{measure}=\frac{2\times P_{recision}\times R_{ecall}}{P_{recision}+R_{ecall}}.$$

In this study, the *P_recision_* and *R_ecall_* values were given by the following equations:

(2) $$P_{recision}=\frac{\sum_{I_{i}\in I}m_{ci}}{\sum_{I_{i}\in I}m_{i}}$$

(3) $$R_{ecall}=\frac{\sum_{I_{i}\in I}m_{ci}}{\sum_{I_{i}\in I}m_{di}}.$$

Here, *m_i_*, *m_ci_*, and *m_di_* refer to the total number of targets, correctly recognised targets, and targets detected by the recognition process for the *i*-th learning image set, respectively. The *F_measure_* value ranges from [0, 1]. A value closer to 1 indicates greater accuracy.

Positional errors (*P_osErr_*) were defined as follows:

(4) $$P_{osErr}=\max\left\{P_{osErr_{1}},\ldots ,P_{osErr_{i}},\ldots ,P_{osErr_{n}}\right\}$$

(5) $$P_{osErr_{i}}=\sqrt{{\left(x_{i}-x_{gti}\right)}^{2}+{\left(y_{i}-y_{gti}\right)}^{2}}.$$

The maximum positional error among *n* targets was used in the evaluation, and each positional error was calculated from the difference between the points detected by the recognition system (*x_i_*, *y_i_*) and the ground truth (*x_gti_*, *y_gti_*). As the proposed system used a moveable camera, we first transformed the positional results from the camera coordinates to world coordinates and then measured the error in millimetres.

The angular errors ($A_{gErr}$) were defined as follows:

(6) $$A_{gErr}=\max\left\{A_{gErr_{1}},\ldots ,A_{gErr_{i}},\ldots ,A_{gErr_{n}}\right\}$$

(7) $$A_{gErr_{i}}=\left|\left(\theta_{i}-\theta_{gti}\right)\left(mod\;360\right)\right|.$$

The maximum angular error among *n* targets was used in the evaluation. As the detection range was from [0, 360), the angular error was given by the difference between the angles detected by the recognition system *θ_i_* and the ground truth *θ_gti_*. This was given by Equation (7).

We set the following order for evaluation: first, the camera distance; second, the *F_measure_*; third, the positional error; and finally, the angular error. Accuracy was determined from the minimum *F_measure_* values and maximum positional error and angular error values. The system would therefore choose the solution based on the FOV size, *F_measure_*, positional error, and angular error, successively. 

For a vision system in pick-and-place tasks, it is important to do recognition as efficient as possible with the guarantee of accuracy. The positional and angular errors can be often tolerated to some extent by the selection of the manipulator though we did not discuss the type of the manipulator in this paper. If the recognition accuracy is high enough, the higher the image capturing efficiency is, the better. Therefore, FOV size and *F_measure_* take the first two priorities for evaluation. If the positional error is too large, the manipulator cannot pick the objects. Therefore, we decided to prioritize positional error over angular error.

#### 2.4.2. Constraints

For the designed vision system to be applied to a pick-and-place task, it is necessary to ensure the minimal performance. In other words, at least the designed vision system could pick up and place the objects without any failure. The constraints are therefore set to guarantee the minimal performance of designed system.

## 3. Methodology

### 3.1. Algorithm Overview

**Figure 1:**
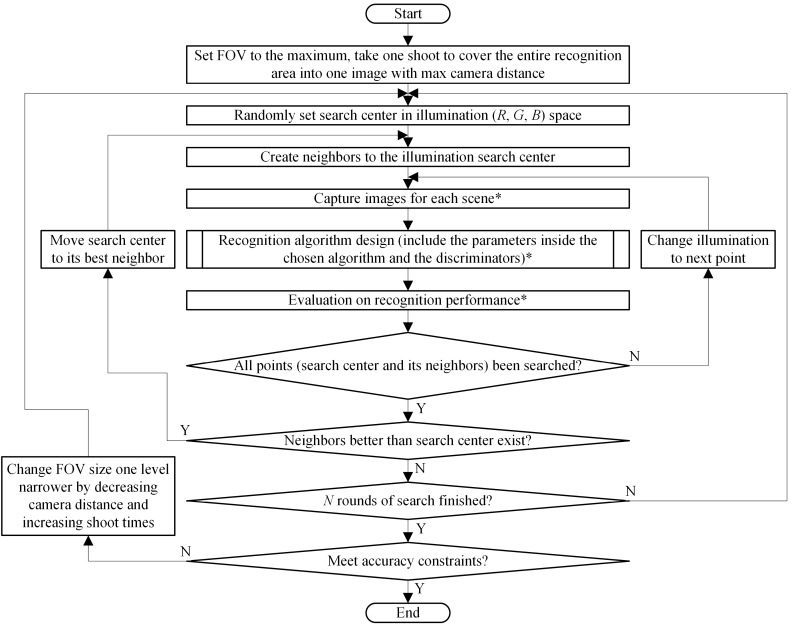
Proposed algorithm to design FOV, illumination and image pre-processing parameters for recognition system. *: The procedures will be skipped if the current selected point has been searched before.

Figure 1 shows the algorithm we proposed to solve the problem formulated in Section 2. In general, we prepared respective optimisations for parameters of the three design factors and arranged them hierarchically.

The system first set the FOV size to its maximum, so that all the target objects could be captured into one image. Based on the multi-start nearest neighbour search, which is discussed in more detail in Section 3.3, the illumination search centre was set randomly to (*Red_i_*, *Green_i_*, *Blue_i_*), and the parameters in recognition algorithm were then designed. After the recognition algorithm design, the system obtained the local solution (*Red_i_*, *Green_i_*, *Blue_i_*, *Height_i_*, *Parameters_Best_*) and its accuracy evaluation (*F_measurei_*, *P_osErri_*, *A_gErri_*) for the corresponding illumination condition. Evaluation was performed repeatedly under neighbour illumination conditions around the selected search centre. After all neighbours were searched, the search centre was moved to its best neighbour until it became the best design. Illumination optimisation was repeated *N* times, yielding *N* local optimal solutions. If solutions meeting the design criteria were found, the system chose the optimal solution among *N* candidates. Otherwise, the system returned to its initial state and narrowed the FOV size by decreasing the camera distance and increasing the shoot time. The methods to apply narrow FOV and estimate FOV size by camera distance are presented in Section 3.2.

### 3.2. FOV Design

**Figure 2:**
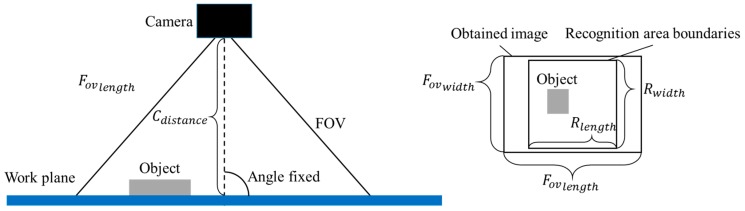
Illustration of the estimation of the FOV size. The positional relation between camera, object(s) and the work plane are shown on left side. The right side shows the length-width ratio of the recognition area and FOV. In the given example, the ratio of FOV is larger than that of the recognition area, which suggests FOV width to represent FOV size.

The FOV is applied to the vision system in the following two ways: first, carry out recognition once with an FOV size and just fit the size of the recognition area and second, carry out recognition by scanning the entire area *n*^2^ times with a FOV of a specific size. Figure 2 shows an example of taking an image of an object placed in the area for recognition. Since the angle between the viewpoint of the camera and the work plane is fixed, which is stated in the preconditions, the FOV size could be easily estimated from the distance between the camera and work plane.

The steps to estimate the FOV size by camera distance are:

*(1) Obtain the mathematical relation between the width of a taken image and camera distance.*

Several images are captured under different camera distances. By adding camera calibrations, the *F_OVwidth_*, or in other words, the distance of *y* direction in the taken images, can be measured in millimetres. Repeating this operation several times, the relations between *F_OVwidth_* and camera distance could be fitted to a linear one:

(8) $$F_{OV_{width}}\left(C_{distance}\right)=a_{width}\times C_{distance}+b_{width}.$$

The *C_distance_* denotes the camera distance, *a* and *b* are coefficients calculated by experimental data.

*(2) Obtain mathematical relation between the length of a taken image and camera distance.*

Similar to FOV width, relations between *F_OVlength_* and camera distance are found using the following expression:

(9) $$F_{OV_{length}}\left(C_{distance}\right)=a_{length}\times C_{distance}+b_{length}.$$

*(3) Choose either length or width to represent the F_OVsize_ based on length-width ratios of the recognition area and captured image.*

(10) $$\frac{F_{OV_{length}}\left(C_{distance}\right)}{F_{OV_{width}}\left(C_{distance}\right)}>\frac{R_{length}}{R_{width}}.$$

*R_length_* and *R_width_* denote length and width of the recognition area, respectively. Equation (10) is the criterion to judge whether to use length or width to represent the size of FOV. Based on the condition of inequality applied in this study, if the length-width ratio of FOV is larger than the ratio of the recognition area, then the width should be selected for calculations in later steps. Otherwise, the length should be chosen.

*(4) Calculate desired FOV size.*

Using either length or width to stand for the size, the desired *F_OVsize_* could be calculated in addition to the size margin and the scan time.

(11) $$F_{OV_{size}}=\frac{R_{size}+\left(\sqrt{S_{time}}-1\right)\times M_{argin}}{\sqrt{S_{time}}}.$$

*R_size_* denotes the length or width of the recognition area, *M_argin_* the margin of the FOV size decided by the maximum size of chosen recognition targets and *S_time_* the total scan time. Here the square root of *S_time_* is used to present the scan time in either the *x* or *y* direction.

*(5) Estimate corresponding camera distance.*

By substitution of the calculated desired FOV size into either Equation (8) or Equation (9), the corresponding camera distance for the desired FOV size could be obtained:

(12) $$C_{distance}=\frac{F_{OV_{size}}-b_{size}}{a_{size}}.$$

Here, *a_size_* and *b_size_* denotes *a_width_* and *b_width_* in Equation (8) or *a_length_* and *b_length_* in Equation (9) depend on the truth or false of Equation (10).

### 3.3. Illumination Design

We selected a random multi-start nearest neighbour search, which is one of metaheuristic method, for optimisation of the illumination strength of red, green, and blue. Due to find constraint satisfaction solutions in limited time, we allowed the system choose search centres randomly, even that may result in different optimums in a fixed condition.

The neighbours were generated by changing the value (adding or subtracting the increments shown in Table 1) of one variable, while holding the others constant. The system then created six neighbours for RGB strength in illumination:

$$\begin{array}{c} N_{eighbors}=\{\left(R+I_{ncrement},G,B\right),\left(R-I_{ncrement},G,B\right),\;\left(R,G+I_{ncrement},B\right),(R,G\\ -I_{ncrement},B),\;\left(R,G,B+I_{ncrement}\right),\left(R,G,B-I_{ncrement}\right)\}. \end{array}$$

### 3.4. Recognition Algorithm Design

The proposed system is capable of automatically selecting threshold values as discriminators for all kinds of recognition objects.

Suppose that a recognition method has a value *E* to evaluate its detections, the higher *E* value indicates the detection is more likely to be a correct one. For a given recognition object, a series of *E* values are used for *n* detections after one recognition.

(13) $$D=\left\{E_{D1},E_{D2},\ldots ,E_{Dn}\right\}.$$

Set ***D*** was then categorised into two sets with the help of ground truth data; ***T*** for correctly detected results, ***F*** for incorrectly detected results:

(14) $$T=\left\{E_{T1},E_{T2},\ldots ,E_{Tm}\right\},$$

(15) $$F=\left\{E_{F1},E_{F2},\ldots ,E_{Fl}\right\},$$

(16) l+m=n.

The threshold *T_h_* was then generated as follows:

(17) $$T_{h}=\max\left\{E_{Ti}|E_{Ti}\in T\cap E_{Ti}<min\left\{E_{Fj}|E_{Fj}\in F\right\}\right\}.$$

The threshold represents the maximal evaluation in the correctly recognised results, and is smaller than the minimal evaluation of the incorrectly recognised results.

As stated before, the optimisation target and corresponding approach rely on the chosen algorithm for recognition. A contour matching method in HALCON library called shape-based matching was utilised as the recognition algorithm in this study.

**Figure 3:**
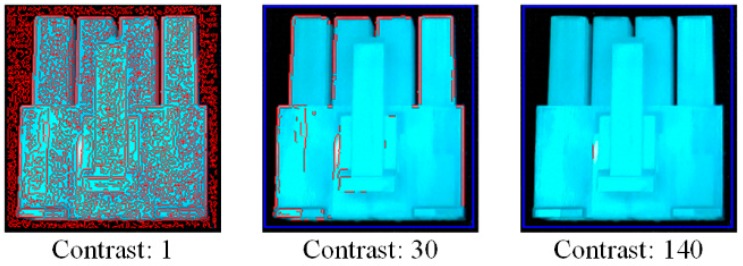
Contour models (marked with red lines) made by different contrast values; the number of contours decrease as the contrast increases. Less contours in a model increase the number of detections that have to be matched and thus result in a longer time taken for matching.

The target parameter of the shape-based matching algorithm to be designed is the contrast value to extract contour models from the templates. Figure 3 illustrates two contour models extracted from different contrast values. A too large contrast value decreases the number of contours in the obtained model to a great extent. Matching with less contours therefore yields more possible candidates, and finally results in longer matching time. If the contour model is decreased to just a short line, the matching time can be infinity.

**Figure 4:**
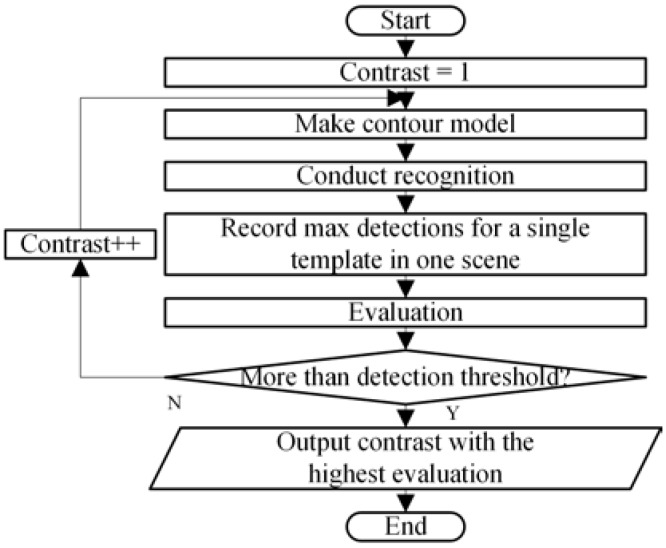
Contour models (marked with red lines) made by different contrast values, the number of contours decrease as the contrast increases. Less contours in a model increase the number of detections that should be matched and thus result in a longer time taken for matching. Algorithm proposed for contrast value design of the chosen recognition algorithm [HALCON (an image processing library of MVTec Company) shape-based matching].

Based on this principle, the design method for the contrast value in shape-based matching is set to traverse all the possible values from the minimum to the maximum and end if the detection number reaches a threshold (Figure 4).

## 4. Evaluation Experiment

### 4.1. Experimental Setup

**Figure 5:**
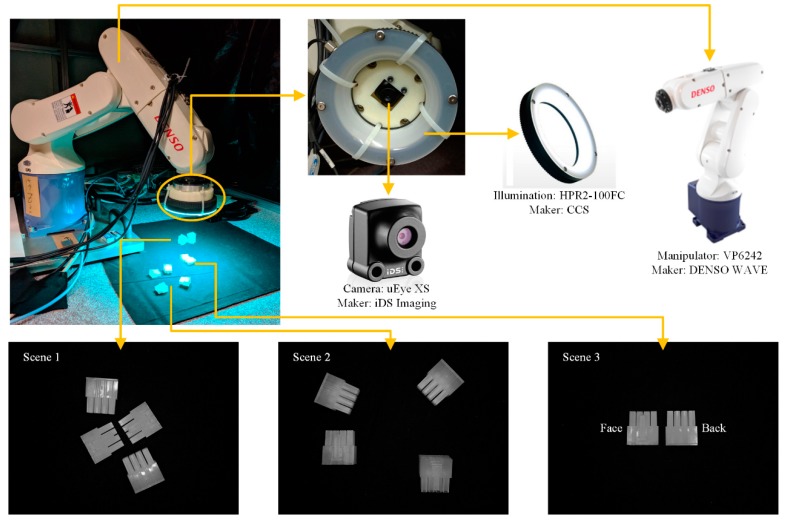
Experimental devices, scenes and recognition targets. Monocular camera and ring-shaped illumination were attached to the end effector of a six-DoF manipulator. Three scenes were prepared on a piece of black cloth; Scene 1 and Scene 2 were for recognition; Scene 3 was for making templates. Two sides of a semi-transparent plastic part (20 mm in length, 20 mm in width, and 8 mm in height) were chosen as the recognition targets.

The experimental environment was an industrial manipulator with six DoFs, a ring-shaped illumination device and an industrial monocular camera (Figure 5). The camera and illumination were mounted on the tips of the manipulator using a 3D-printed joint. The processor was an Intel Core i5-5300U@2.30 GHz.

To reflect potential applications, we chose the two sides of a semi-transparent plastic part (Figure 5) as the recognition target. Different from its side at the rear, the face side had a convex structure in the middle. The following constraints were applied: an *F_measure_* score no less than 1; a positional error no more than 3 mm; and an angular error no more than 5°.

Three scenes with different functions were arranged on a piece of black cloth below the manipulator (Figure 5). In order to prevent overfitting, two scenes were prepared for recognition. Every time the FOV or illumination changed, the templates were updated; a scene for updating the templates was therefore required. Scene 1 and Scene 2, with two face and two rear side objects in each, were set up for recognition. Scene 3, with a face side and a rear side object, was set up to create templates.

Two different plans for FOV were given to our system: one was to shoot once with a wide FOV at a camera distance of 158 mm, and the other was to shoot four times with narrow FOVs at a camera distance of 105 mm.

We controlled the colour channels utilised in both recognition and illumination, and created three experimental conditions. Recognitions were conducted with greyscale images in Condition I and II, while R-channel images were used in Condition III. The details are listed below. On the other hand, illuminations were changed from only G channel in Condition I, and changed from RGB three channels in Condition II and III. Details of the conditions based on which each experiment was conducted are listed in Table 2.

The reason why G illumination was chosen in Condition I is that it is considered to influence the brightness in the obtained images to the greatest extent. Therefore, the dimension of illumination was reduced to a great extent, and the increment of illumination strength was set to 1 in Condition I.

For all aforementioned conditions, we manually measured the ground truth data. For the illumination variables, 16 local optimisation searches were performed. The maximum detection to end contrast value search was set to 4.

### 4.2. Results

**Figure 6:**
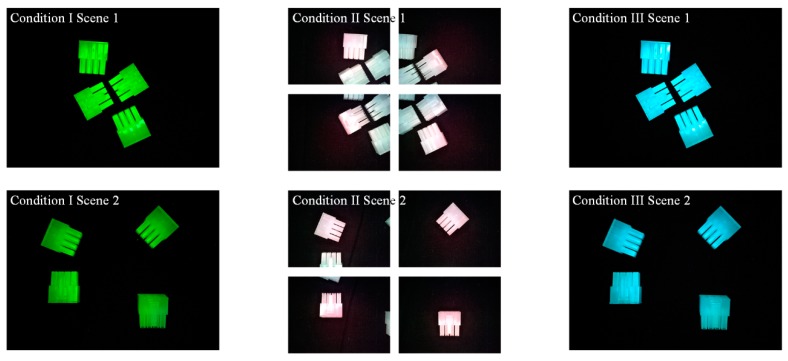
Optimal illumination and FOV conditions designed for the three conditions. Condition I: 1 shoot under strong green illumination. Condition II: four shoots under illumination with red component relatively higher. Condition III: 1 shoot under strong green and blue illumination.

The best-three solutions and their evaluations of the three conditions are presented in Table 3, Table 4 and Table 5 and the images taken under the optimal parameter sets are shown in Figure 6.

Proper design could not be achieved with either with one shot or four shots when tuning illumination from only G component in Condition I. The optimal design was realised with a 79 in green illumination, four shots and a contrast value of 3, which provided a 0.93 *F_measure_*, about 0.6 mm maximum positional error, and 2.1° maximum angular error.

The optimal design of Condition II corresponded to a (195, 120, 75) illumination RGB strength, four shoots and contour models generated by a contrast value of 4. This set up resulted in an *F_measure_* value of 1, a maximum positional error of about 0.6 mm, and a 3.1° maximum angular error.

Replacing the greyscale images with R-channel images, suitable designs were found only with one shoot. The optimal design was (195, 120, and 75) in illumination RGB, 11 in contrast value, and with one shoot. Its evaluation showed an *F_measure_* of 1, about 0.3 mm in maximum positional error, and 0.4° in maximum angular error.

## 5. Discussion

Generally speaking, designs under the accuracy constraints, that is, an *F_measure_* of 1 and no more than 3 mm and 5° in positional and angular errors were found in both conditions of illumination tuned from RGB channels. This finding proved that our system is capable of tuning parameters for a vision system used in pick-and-place tasks.

Comparing the results of the first two conditions, designs under accuracy constraints were found when illumination was tuned from RGB, while no proper design was found with an *F_measure_* of 1 with illumination tuned only from G. In both conditions, the input images were of the greyscale type, which indicated that although a vision system finally converts colour images into grey, it is still essential to tune the illumination based on the three RGB channels.

On the other hand, from the results of Condition II and Condition III, it was found that using R-channel images could provide better performance in recognition than greyscale ones. Designs with one shot and a wide FOV were found in Condition III, while a narrow FOV was designed with four shoots in Condition II.

**Figure 7:**
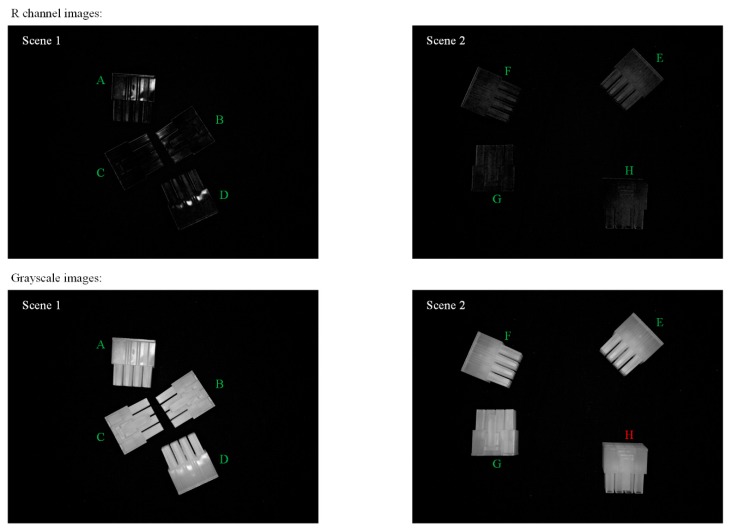
R-channel images, greyscale images for the two scenes under the optimal design of Condition III [illumination RGB (red, green and blue) equals to 15, 225 and 240, with a wide FOV], and their recognition results. Objects labelled in green were those that could be correctly recognised, while the red one could not be recognised.

In order to further discuss the effects of R-channel images, the R-channel images for the two scenes under the optimal design of Condition III (illumination RGB equals to 15, 225, and 240, 1 shoot) were extracted. We processed the two figures with greyscale; both R-channel and greyscale figures are shown in Figure 7. Moreover, to confirm that the R-channel images perform better than greyscale images under the same situation, an additional design was implemented with greyscale images, as shown in Figure 7. Results showed that the optimal design with the greyscale images could only provide an *F_measure_* of 0.93.

To our human eye, it is obvious that the greyscale images are easier to recognise. However, in a vision system, R-channel images are recognised with a higher recognition accuracy. The probable reason might be that in a sufficiently bright image, the noise is also enlarged to a great extent. Vision systems do not detect a picture as humans do; these systems read the limited features in the form of mathematical values in matrixes instead. When the noise is so large that it obscures the useful information indicated in these matrixes, judgements made by the system could be flawed. From this point of view, the key to a ‘clear’ image for vision systems is that these images must contain little but important information. As an example, though the R-channel images in Figure 7 were really dark, the contours of each object could still be seen clearly. The great contrast between contours and background therefore make the images ‘clear’. In some ways, image pre-processing is just a method to serve the vision systems with ‘clearer’ images.

**Figure 8:**
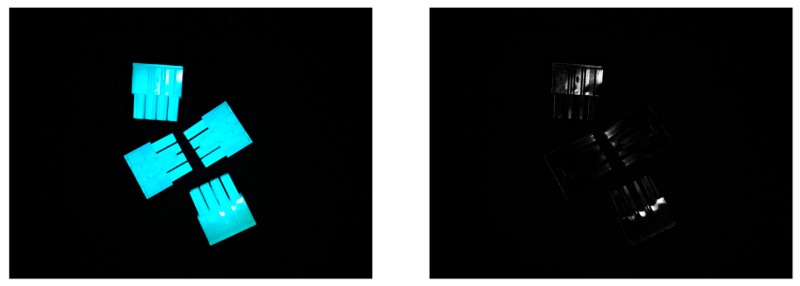
An image taken with no red component in illumination (illumination RGB were set to 0, 225, and 255, respectively), and its R-channel image.

Moreover, illumination in the designs with high evaluations showed no relations to each other with greyscale images input, while a clear pattern was discovered in the circumstance of R-channel images. Based on Table 4, illuminations of the best-three designs were found with low red illumination (under 50), high green illumination (near 225), and relatively high blue illumination (from 150 to 240). Generally speaking, tuning green and blue illuminations is not effective when the image can only be seen using a red channel. However, Figure 8 shows that even with no red illumination, the objects are visible in the R-channel image. The probable reason may be that the RGB tuned from the illumination side is not the same as the RGB information contained in an image. Because of the wavelength of the illumination device or some reflections, the G and B components could still influence the R-channel image to some extent. Actually, such an influence eventually resulted in ‘clearer’ R-channel images compared with the greyscale ones. The illumination pattern found in Condition III also confirmed the importance of green and blue illumination. In addition, patterns of illumination indicated that relations might exist between the recognition performance and its illumination conditions, which give rise to possibilities for the application of other optimisation methods.

Nevertheless, the experiment was limited under the environment we prepared. We could only state that R-channel image could provide better recognition accuracy under the experimental settings. We cannot affirm that whether this phenomenon could be discovered with other recognition targets, or by recognition with other algorithms. To better explain it, further experiments will be required.

## 6. Conclusions

In this study, we proposed an automated design approach for vision systems in pick-and-place tasks. The vision system design was first formulated as a parameter optimisation problem and then solved in an experiment-based approach with a hierarchical algorithm. Rather than seeking a suitable parameter set randomly in the solution space, the proposed algorithm separates and sets hierarchies for each optimisation based on the design factors. As one of the uncertainties from the real world, the influence of colour on the recognition performance of the designed vision systems was also investigated through experiments in this research.

It could be seen through the experiments that the proposed system was able to design a vision system with a 100% recognition rate, and a positional and angular error of 0.32 mm and 0.4°, respectively. When using greyscale images for recognition, G illumination resulted in an *F_measure_* of only 0.93, which proved the necessity for colourful illumination. Consequently, when RGB illumination was used, designs with R-channel images used only one shot, which indicates that R-channel images provide better recognition accuracy than the greyscale ones.

In future work, from the viewpoint of robustness, it is necessary to improve the prevention against overfitting by increasing the number of scenes for recognition and include the measurement of overfitting in the evaluation of the designed vision system. Aiming to take out better solutions, the selection of recognition algorithm should also be included into the design process. Additionally, further research could be conducted on searching more appropriate optimisation methods, for example, neural networks or genetic algorithms, to provide better solutions that are less time-consuming for the vision system design problem.

## Figures and Tables

**Table 1:** List of design variables.

Design Factor	Name	Description	Range
FOV	Shoot time	Number of images required in one recognition for the entire area	1, 4, …, *n*^2^
	Camera distance	Represents FOV size	Determined by shoot time
Illumination	Light strength (Red)	Strength of red component in illumination	[0, 255]
	Light strength (Green)	Strength of green component in illumination	[0, 255]
	Light strength (Blue)	Strength of blue component in illumination	[0, 255]
Recognition algorithm	Discriminator	Thresholds for classifying different kinds of recognition objects	(0, 1)
	Contrast	Contrast value to extract contour model from template	[0, 255]

**Table 2:** Experimental conditions.

Condition	Illumination Channel(s)	Increment of Illumination Parameter(s)	Recognition Image(s)
I	G only	1	Greyscale
II	RGB	15	Greyscale
III	RGB	15	R-channel

**Table 3:** Best-three designs of Condition I.

Rank	R	G	B	FOV	Contrast	*F_measure_*	Positional Error (mm)	Angular Error (°)
1	0	232	0	wide	1	0.93	0.64	2.1
2	0	79	0	narrow	3	0.93	0.58	3.4
3	0	84	0	narrow	3	0.86	0.35	3.6

The results were ranked by their evaluations; higher rank represents better evaluation. A wide FOV denotes one shot at a camera distance of 158 mm, and a narrow FOV denotes four shots at a camera distance of 105 mm.

**Table 4:** Best-three designs of Condition II.

Rank	R	G	B	FOV	Contrast	*F_measure_*	Positional Error (mm)	Angular Error (°)
1	195	120	75	narrow	4	1.00	0.60	3.1
2	240	45	75	narrow	3	1.00	0.92	3.0
3	105	30	150	narrow	4	1.00	1.15	2.9

The results were ranked by their evaluations; higher rank represents better evaluation. A wide FOV denotes one shot at a camera distance of 158 mm, and a narrow FOV denotes four shots at a camera distance of 105 mm.

**Table 5:** Best-three designs of Condition III.

Rank	R	G	B	FOV	Contrast	*F_measure_*	Positional Error (mm)	Angular Error (°)
1	15	225	240	wide	11	1.00	0.32	0.4
2	0	225	150	wide	11	1.00	0.50	0.4
3	45	225	210	wide	9	1.00	0.62	0.4

The results were ranked by their evaluations; higher rank represents better evaluation. A wide FOV denotes one shot at a camera distance of 158 mm, and a narrow FOV denotes four shots at a camera distance of 105 mm.
